# Dose optimization for CT scans of the temporal bone using spectral shaping tin filter

**DOI:** 10.1002/acm2.70359

**Published:** 2025-12-29

**Authors:** Alexander von Hessling, Carlos Vinícius Gomes, Pia Niederau, Thiago Lima, Justus Roos, Natalia Saltybaeva

**Affiliations:** ^1^ Department of Radiology and Nuclear Medicine Luzerner Kantonsspital, University Teaching and Research Hospital University of Lucerne Lucerne Switzerland

**Keywords:** CT, radiation dose, tin filter

## Abstract

**Background:**

Computed tomography is a highly effective diagnostic tool for evaluating pathological conditions of the temporal bone. However, the relatively high radiation doses pose risks to radiosensitive organs, making dose optimization essential in accordance with the ALARA principle. Recent technological advances, such as tin‐based photon‐shaping filters, have enabled significant dose reduction in both dual‐ and single‐energy CT without compromising image quality.

**Purpose:**

To evaluate the impact of a spectral shaping tin filter on image quality and radiation dose in temporal bone CT examinations.

**Methods:**

Thirty‐six patients undergoing temporal bone CT were retrospectively analysed. Group A (*n* = 13) underwent standard CT, while Group B (*n* = 23) underwent CT examination using 150 kVp beam with additional tin filtration. Images were reconstructed with model‐based iterative reconstruction (ADMIRE, level 3) and assessed by two neuroradiologists using a 3‐point Likert scale. Radiation dose was quantified via CTDIvol, DLP, and eye lens dose using Monte Carlo simulations.

**Results:**

Both protocols provided diagnostic‐quality images. The protocol with tin filter achieved slightly superior image quality (median scores: overall 3.00 vs. 2.88, contrast 3.00 vs. 2.96, resolution 2.58 vs. 2.53; *p* < 0.05) with substantial inter‐rater agreement (*κ* = 0.75–0.97). Mean CTDIvol and DLP decreased from 59.1 ± 12.1 mGy and 551.8 ± 226.8 mGy×cm (Group A) to 39.9 ± 1.7 mGy and 302.4 ± 33.8 mGy×cm (Group B) (*p* < 0.001). Eye lens dose was reduced by 34% (54.7 ± 10.9 mGy vs. 36.4 ± 2.2 mGy, *p* < 0.001).

**Conclusions:**

Incorporating a tin filter into temporal bone CT significantly reduces radiation exposure, including lens dose, without compromising—and in some cases slightly improving—image quality. Routine implementation of spectral shaping may enhance patient safety in clinical practice.

## 1 INTRODUCTION

Computed tomography (CT) is one of the most powerful diagnostic techniques for evaluating a wide range of pathological conditions. Since the advent of multidetector technology, CT has become a major imaging modality for visualizing the fine anatomical structures of the temporal bone, owing to its high spatial resolution.^1^ However, radiation exposure in this examination remains a significant concern due to the relatively high doses required to achieve such resolution and the presence of radiosensitive organs, such as the eye lenses, within the scanned volume.^2^, ^3^ Therefore, particular attention in temporal bone CT imaging must be given to patient dose optimization, in accordance with the “as low as reasonably achievable” (ALARA) principle.

In recent years, several technical innovations have been introduced to reduce radiation dose in CT, including automatic tube current modulation, automatic voltage selection, and iterative image reconstruction.^4^, ^5^, ^6^ Another such innovation is spectral shaping, typically achieved using a tin (Sn) filter mounted in front of the x‐ray tube. Initially, the Sn filter was implemented to improve spectral separation in dual‐energy CT (DECT), where it is usually combined with high tube voltages of 140–150 kV to remove low‐energy photons from the x‐ray spectrum allowing for significant dose reduction without compromising the image quality. Additionally, the Sn filter can be used to maintain adequate spectral separation when employing 100 kV for the low‐potential tube instead of 80 kV, which also enables dual‐energy imaging in larger patients where 80 kV acquisitions may not be practical.^7^, ^8^ More recently, it has been demonstrated that the Sn filter can also be used in single‐energy CT, enabling more efficient x‐ray beam utilization and thus radiation dose reduction.^9^

The studies have investigated the benefits of spectral shaping for various clinical applications.^10^ Tin filters have been used for ultra‐low dose chest CT protocols, particularly during the COVID‐19 pandemic.^11^, ^12^ They have also been used for coronary artery calcium scoring CT and to detect urinary tract stones or osteolytic lesions in multiple myeloma.^13^, ^14^, ^15^

Although several groups have evaluated shaping filters in temporal bone CT using phantom models^16^ and cadavers,^17^ studies involving real patients remain limited.^18^ In this study, we compared a standard temporal bone CT protocol with one incorporating a tin shaping filter in a clinical patient population. Unlike previous investigations, we assessed not only overall radiation exposure but also the dose to sensitive organs within the scanned region.

## 2 MATERIALS AND METHODS

### 2.1 Patient population

This study was approved by the local institutional review board, and the requirement for written informed consent was waived.

CT examinations were performed using a Somatom X.ceed scanner (Siemens Healthineers, Erlangen, Germany). A total of 36 patients (mean age 52.6 ± 18.6 years; range 18–81 years) who underwent temporal bone CT between October 2022 and August 2025 were included.

Patients were divided into two groups: Group A included 13 patients (4 males, 9 females) who underwent CT using the standard temporal bone protocol, utilizing CareDose4D which selects kV and mAs individually for each patient, based on the attenuation from the scout image. Group B included 23 remaining patients (10 males, 13 females), who underwent CT after protocol optimization, which involved the addition of a shaping filter and an increase in tube potential to 150 kVp. Detailed parameters for both protocols can be found in Table 1.

**TABLE 1:** Protocol parameters used for the temporal bone CT examination.

CT Protocol	Group A: Temporal bone conventional	Group B: Temporal bone with Sn filter
CareDose4D	ON	OFF
Tube voltage, kV	CareDose4D	150 (fixed)
Additional filtration	–	0.6‐mm tin (Sn) filter
Quality ref. mAs	350	300
Pitch	0.55	0.55
Beam width, mm	19.2	19.2
Kernel	Hr68w	Hr68w
Reconstructed slice thickness, mm	0.4	0.4
Iterative reconstruction	ADMIRE 3	ADMIRE 3

### 2.2 Image quality

All CT images were reconstructed using the advanced model‐based iterative reconstruction technique (ADMIRE, Siemens) at a strength level of 3. Image quality was independently evaluated by two board certified neuroradiologists with 22 and 8 years of experience in imaging. A 3‐point Likert scale was used (1 = poor, 2 = good, and 3 = excellent) to assess three parameters: overall image quality, contrast and resolution.

### 2.3 Radiation dose

Radiation exposure was assessed using the volumetric CT dose index (CTDIvol) and dose‐length product (DLP) retrieved from the patient dose reports.

Additionally, the dose received by the eye lens was assessed for each patient scanned with and without the spectral shaping filter. For this purpose, the Monte Carlo (MC) simulation–based calculations were performed using the VirtualDose tool (Virtual Phantoms Inc., Albany, New York, USA). The 50th percentile RPI male and female phantom models representing average adult male and female were used for MC calculations. Protocol‐specific parameters for organ dose assessment were retrieved from the DICOM headers of the CT images for each patient.

### 2.4 Statistical analysis

Differences in subjective image quality between groups A and B were assessed using the Mann–Whitney U‐test. Inter‐rater reliability was evaluated using Cohen's kappa coefficient, with values interpreted as follows: <0.20 = slight agreement, 0.21–0.40 = fair, 0.41–0.60 = moderate, 0.61–0.80 = substantial, and > 0.80 = almost perfect agreement [14]. Differences in radiation dose metrics (CTDIvol and DLP) as well as the difference in eye lens doses were assessed using the Student's *t*‐test. Statistical analyses were performed using Python (version 3.10; Python Software Foundation).

## 3 RESULTS

### 3.1 Image quality

The image quality analysis performed by two independent radiologists has shown that both CT protocols with and without shaping photon filtration provide high diagnostic images quality for patients with indications for a high‐resolution petrous bone imaging. Typical clinical settings were patients with hearing loss and suspected otosclerosis, cholesteatoma, defects in the ossicular chain, or inner ear abnormalities. The subjective image quality assessment performed using the 3‐point Likert scale was used (1 = poor, 2 = good, and 3 = excellent) for overall image quality, contrast and resolution have shown that the median scores for the conventional temporal bone CT protocol without spectral shaping filter were 2.88 (overall), 2.96 (contrast), and 2.53 (resolution), while the corresponding scores for protocol with additional tin filter were 3.00, 3.00, and 2.58, respectively. Thus, the protocol with tin filter has demonstrated slightly superior image quality, and this difference was statistically significant (*p* = 0.0076).

**FIGURE 1:**
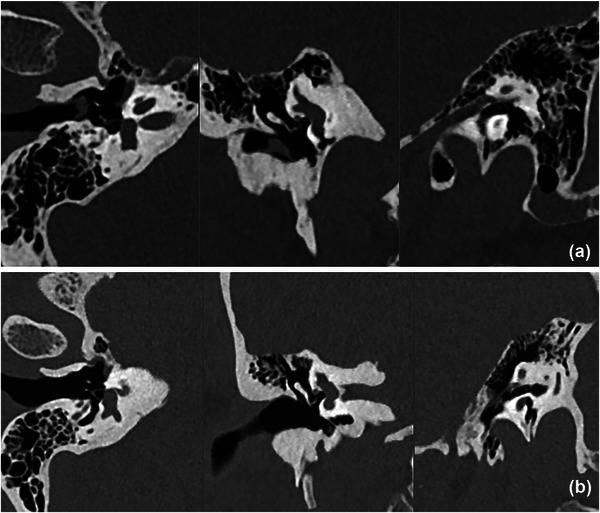
Para‐sagittal, para‐coronal and para‐axial CT image of the patients scanned with (a) shaping spectral tin filter and (b) scanned without tin filter, reconstructed with 0.4 mm slices (without interval), ADMIRE iterative reconstruction strength 3 and HR68 kernel.

Inter‐rater reliability, assessed using Cohen's kappa coefficient, showed substantial agreement between the two radiologists: 0.92 for overall image quality, 0.97 for contrast, and 0.75 for resolution. Figure 1 shows an example of the images acquired using the protocol with and without tin filter.

### 3.2 Radiation dose

**FIGURE 2:**
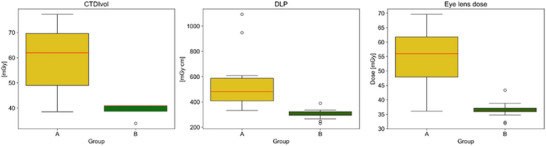
Comparison of CTDIvol, DLP, and eye lens dose between data acquired without tin filter (Group A) and with tin filter (Group B). Box plots show the distribution of the data, with the red line indicating the mean, the box spanning the 25th–75th percentiles (Q1–Q3), whiskers extending to 1.5 × IQR, and points beyond the whiskers representing outliers.

The mean CTDIvol values were found to be 59.1 ± 12.1 and 39.9 ± 1.7 mGy for protocol without and with tin filter, respectively. The mean DLP, calculated as a product of CTDIvol and the scan length was equal to 551.8 ± 226.8 and 302.4 ± 33.8 mGy×cm for protocol without and with tin filter, respectively. This difference was statistically significant (*p* < 0.001). The mean eyes dose for patients scanned with tin filter was found to be 36.4 ± 2.2 mGy with the range from 31.8 to 38.7 mGy, while the mean eye lens dose for patients scanned with conventional CT protocol varied from 36.1 to 69.6 mGy with the mean values of 54.7 ± 10.9 mGy. Thus, the relative eye lens dose reduction achieved due to the additional spectral filtering was 34%. Statistical analysis showed no significant difference between groups regarding age (p= 0.5514). In contrast, highly significant differences were found for all dose‐related parameters: CTDIvol (p< 0.001), DLP (p< 0.001), and eye lens dose (p< 0.001). Radiation dose parameters for each group, without the filter (Group A) and with the tin filter (Group B), are shown in Figure 2.

## 4 DISCUSSION

Temporal bone CT remains an essential imaging tool for the evaluation of otologic and skull base pathologies due to its superior spatial resolution. However, its use raises significant concerns regarding radiation exposure, particularly because the eye lenses are positioned within the primary beam and are highly radiosensitive. Consequently, both the overall patient dose and the dose delivered to sensitive organs warrant careful consideration. Our study demonstrates that incorporating a spectral shaping tin filter into temporal bone CT protocols can achieve a substantial reduction in radiation exposure, as evidenced by decreases in both CTDIvol​ and estimated organ doses, without compromising diagnostic image quality. The CTDIvol​ reduction observed in this study is in line with similar research for other indications, such as calcium scoring CT and urinary stone imaging, where reductions from 29% to 75% and from 30% to 60% have been reported, respectively.^19^, ^20^ In a similar study performed by Kim and Jeon (2018)^18^ a 150 kVp Sn‐filtered temporal bone CT protocol was reported to reduce the effective dose by nearly 70 % compared to the conventional 120 kVp protocol, which is greater than the reduction observed in our study. However, they did not report any significant improvement in image quality with the Sn‐filtered protocol. In contrast, Grunz et al.^17^ who investigated image noise at low, intermediate, and high dose levels in cadaver heads, demonstrated that a 150 kVp tin‐filtered protocol provided superior image quality compared with the 120 kVp protocol without additional filtration. In contrast to previous studies, the present work not only compared standard exposure parameters, such as CTDI and DLP, but also assessed the radiation dose delivered to the eye lens for both investigated protocols. This is especially important given that even relatively low radiation doses to the lens can increase the risk of radiation‐induced cataracts, as highlighted in recent epidemiological studies and reflected in the International Commission on Radiological Protection's revised dose limits for the eye lens.^21^ Our study demonstrated that the dose to the eye lens can be reduced by 34% on average using a 150 kVp beam with additional tin filtration (Group B) instead of the conventional protocol with adaptive kVp and no shaping filtration (Group A). Importantly, these dose savings were accomplished without compromising spatial resolution or contrast — critical parameters for the evaluation of fine temporal bone structures. In fact, in some cases, image quality metrics were slightly improved, with higher average values for the image quality score in Group B.

These findings align with prior phantom‐based investigations showing that tin filtration can achieve ultra‐low‐dose acquisitions for various anatomical regions, but they extend the evidence to a clinical patient population for temporal bone imaging. The eye lens dose reduction achieved here is particularly significant for patients requiring repeated temporal bone imaging, such as those with chronic ear disease, congenital malformations, or postoperative follow‐up, where cumulative dose to radiosensitive organs is a long‐term concern.

A limitation of this study is the relatively small dataset, the use of a 3‐point Likert scale for subjective image quality assessment, and the limited number of questions posed to the radiologists. Once it was evident that the new protocol using the tin filter performed no worse than the standard protocol while achieving dose reduction, we ethically discontinued scanning additional patients without filtration (Group A). Despite these limitations, the results indicate that the new protocol using Sn filtration, which allows for significant dose reduction, is not inferior to the standard protocol without additional filtration. Another limitation is that organ dose calculations did not take individual patient position or habitus into account; therefore, these estimates do not represent individual patient risk and should be interpreted only as comparative metrics between protocols.

## 5 CONCLUSION

In summary, our findings provide strong evidence that tin filter–based spectral shaping is a valuable tool for dose optimization in temporal bone CT, particularly for protecting the radiosensitive eye lens. The substantial reduction in radiation exposure, along with maintained or slightly improved image quality, supports the routine implementation of this approach in clinical practice. Given the growing awareness of lens radiosensitivity and the frequency of temporal bone imaging in certain patient populations, widespread adoption of tin filtration protocols could represent a meaningful step forward in patient safety without compromising diagnostic accuracy.

## AUTHOR CONTRIBUTIONS

Data collection and analysis were performed by Carlos Vinícius Gomes and Natalia Saltybaeva. Image analysis was conducted by Pia Niederau and Alexander von Hessling. Thiago Lima and Justus Roos coordinated the project and provided conceptual guidance. The manuscript was written by Alexander von Hessling and Natalia Saltybaeva, with all authors contributing to revisions and improvements. Natalia Saltybaeva served as the corresponding author and is responsible for overall project oversight.

## GENERATIVE AI AND LARGE LANGUAGE MODELS

No generative AI tools or large language models were used in the preparation of this manuscript.

## CONFLICT OF INTEREST STATEMENT

The authors declare no conflicts of interest.
